# Development and implementation of a significantly low-cost 3D bioprinter using recycled scrap material

**DOI:** 10.3389/fbioe.2023.1108396

**Published:** 2023-04-07

**Authors:** Jaciara Fernanda Gomes Gama, Evellyn Araujo Dias, Rosângela Marques Gonçalves Aguiar Coelho, André Maia Chagas, José Aguiar Coelho Nt, Luiz Anastacio Alves

**Affiliations:** ^1^ Laboratory of Cellular Communication, Oswaldo Cruz Institute, Oswaldo Cruz Foundation, Rio de Janeiro, Brazil; ^2^ Sussex Neuroscience, School of Life Sciences, University of Sussex, Brighton, United Kingdom; ^3^ TReND in Africa, Brighton, United Kingdom; ^4^ Biomedical Science Research and Training Center, Yobe State University, Damaturu, Nigeria; ^5^ National Institute of Industrial Property- INPI and Veiga de Almeida University, Rio de Janeiro, Brazil

**Keywords:** 3D bioprinter, biomolecules, biotechnology, 3D print, low-cost, scrap-metal

## Abstract

The field of 3D bioengineering proposes to effectively contribute to the manufacture of artificial multicellular organ/tissues and the understanding of complex cellular mechanisms. In this regard, 3D cell cultures comprise a promising bioengineering possibility for the alternative treatment of organ function loss, potentially improving patient life expectancies. Patients with end-stage disease, for example, could benefit from treatment until organ transplantation or even undergo organ function restoration. Currently, 3D bioprinters can produce tissues such as trachea cartilage or artificial skin. Most low-cost 3D bioprinters are built from fused deposition modeling 3D printer frames modified for the deposition of biologically compatible material, ranging between $13.000,00 and $300.000,00. Furthermore, the cost of consumables should also be considered as they, can range from $3,85 and $100.000,00 per gram, making biomaterials expensive, hindering bioprinting access. In this context, our report describes the first prototype of a significantly low-cost 3D bioprinter built from recycled scrap metal and off-the-shelf electronics. We demonstrate the functionalized process and methodology proof of concept and aim to test it in different biological tissue scaffolds in the future, using affordable materials and open-source methodologies, thus democratizing the state of the art of this technology.

## 1 Introduction

Tissue engineering is an extensive study and promising biotechnology field, allowing for the design of tissues from cells, matrices and other materials (Salgado et al., 2013; Shah et al., 2022), performing well in translational research, such as the clinical transplantation of bioengineered corneas (González-Andrades et al., 2017). These findings have stimulated the search for new methodologies to assess bioengineered tissues and transpose them to several human tissues and organs (Harrison et al., 2014). In this regard, current technological developments, such as artificial intelligence, machine learning technology (Ng et al., 2020a; Yu and Jiang, 2020), 3D printing, nano- and biotechnology, are now leading the world into the fourth industrial revolution, characterized by faster than ever development, process automation and impacts in most, if not all industries (The Fourth Industrial Revolution, 2022). Given this revolution is powered by many open-source tools and technologies, enabling distributed manufacturing, customization, local repairs and lowering entry costs (Maia Chagas, 2018), it stands to reason that low- and middle-income countries may be active participants in this revolution, especially in the health technology area. However, biology research equipment and medical devices continue to present high price tags and are hard to obtain, even when funds are available, due to issues related to the geographical distribution of suppliers, custom taxes. In this context, we have developed an open source microextrusion bioprinter for tissue engineering using discarded computer materials, medical devices, and off-the-shelf electronics.

Bioprinters have displayed strong development in recent decades, and the high demand for organs and tissues has increased the need for large-scale bioprinting production to aid patients with organ malfunctions or in drug delivery research. Several bioprinters are commercially available and/or described in the literature. However, they are costly, as bioinks are proprietary and not easily accessible, making bioprinting difficult and not as widely available as should be. Table 1 reports cost estimations of previously reported low-cost bioprinters and our prototype created from different materials. Current models are used to produce *in vitro* tissue and organ frameworks, leading to extremely precise material deposition and enabling architectures very similar to *in vivo* conditions (Ozbolat, 2015). Current bioprinting technologies include inkjet, laser-assisted (Oliveira et al., 2017), microextrusion 3D bioprinting (Bishop et al., 2017), droplet-by-droplet technology (Ng et al., 2021) and vat polymerization-based bioprinter (Ng et al., 2020b). Table 2 shows advantages and shortcomings of the main techniques. Different materials deposited by bioprinters are collectively called bioinks, and include live cells, nanocompounds, collagen, alginate and peptide-based hydrogels, forming structure capable of projecting themselves into previously established conformations (Ozbolat, 2015). Bioprinters need to work at biocompatible temperatures, in contrast to fused deposition modeling (FDM) 3D printers, where the printing nozzle heats up to about 200°C (making the printing material soft and easy to extrude, requiring different strategies to maintain high resolution as well as cell viability, in bioink (Jiang et al., 2019).

**TABLE 1:** Bioprinters built with low-cost parameters.

References	Price	Based on		Extrusion nozzles	Country
Our prototype	∼$ 260,00* or less than * $120,00 if recycled metal scrap material is used			DVD-ROM drivers to get the mechanics and building a stepper motor injection module	Brazil
Ioannidis et al. (2020)	∼$230,00	3D printer Anet A8		1 (Two different nozzle diameters were tested)	Greece
Wagner et al. (2021)	∼$200,00	DVD-drive components (using 3D printed parts by Anet A8)		2 (One nebulizer extrudes the hardening solution)	Austria
Kahl et al. (2019)	∼$170,00	Anet A8 Desktop 3D Printer Prusa i3		1	Germany
Krige et al. (2021)	$300,00	Prusa i3 MK3 3D printer		1	Sweden
Sanz-Garcia et al. (2020)	∼$70,00	Witbox2; RepRap BCN3D+; and Sigma 3D printer		1	Finland

**TABLE 2:** Technologies applied to 3D bioprinting: general pros and cons.

Types of bioprinter	Sub-types	Resolution	Cell viability	Advantages	Disadvantages	Ref
Inkjet mechanism	Thermal; Piezoelectric	10–200 µm	∼85%–90%	Fast Speed; Low cost; High resolution; Precise deposition Wide availability	Easy to clog; Works only on low viscocity bioinks;	Murphy and Atala, (2014); Saunders and Derby, (2014); Li et al. (2016); Kačarević et al. (2018); Lee et al. (2018)
Extrusion mechanism	Piston; Screw; Pneumatic	5–400 µm	∼40%–95%	Support high cell density; Potential for multi-material bioprinting; Relatively good resolution Easy to implement; Medium cost; High versatility and feasibility	Low resolution; Low speed Shear stress may impact cell viability	Murphy and Atala, (2014); Saunders and Derby, (2014); Li et al. (2016); Kačarević et al. (2018); Lee et al. (2018)
Laser-assisted mechanism	Not applicable	>20 µm	>95%	High resolution; Nozzle-free; High control of bioink droplets	High cost; Medium speed; Time consuming; Low stability and scalability	Murphy and Atala, (2014); Saunders and Derby, (2014); Li et al. (2016); Kačarević et al. (2018)
Vat Photopolymerization	Stereolithography; Digital Light Processing	∼1.2–300 µm	∼40%–95%	Low cost; Nozzle-free; Fast speed; Highest fabrication accuracy; Absence of shear stress; No limitation on bioink viscosity	Works only on photopolymer bioinks; Lack of biocompatibility Risk of damage caused by UV light to cell DNA; Cytotoxicity	Murphy and Atala, (2014); Li et al. (2016); Kačarević et al. (2018); Lee et al. (2018)

All current low-cost, extrusion based models either use a commercially available 3D printer as base or employ it to create bioprinter parts (Kahl et al., 2019; Ioannidis et al., 2020; Sanz-Garcia et al., 2020; Krige et al., 2021; Wagner et al., 2021). The need for a 3D printer is, however, not included in the prices displayed in Table 1, making the cost of those solutions underreported. In our current design, the basic costs are more attractive than the proposed costs of different low-cost bioprinters in the literature, including some pieces beyond the scrap material (Table 3). It is important to note that, even with the low cost of the prototype proposed here, the ability to create more complex prints is still maintained, including only one extrusion nozzle, while more expensive bioprinters normally have more nozzles (Table 4).

**TABLE 3:** Components used to build the prototype described here.

Component	Detailed description
Insulin syringe with fixed needle	1 mL capacity, 8 mm × 0.30 mm needle size
06FR caliber urethral probe	40 cm in length
Hypodermic needle of head print	25 mm × 0.70 mm
Syringe with a “Luer lock” needle fitting	3 mL capacity
2-component epoxy glue	Set time of 30 min
A 5 cm piece of mild steel wire	Approximately 0.5 mm in diameter

**TABLE 4:** Comparative list of commercially available bioprinters per mechanism and customized prototypes prices.

Model	Price	Technology	Country	References
TissueStart—TissueLabs	∼$7.999,00	Syringe-based extrusion	Brazil	https://www.tissuelabs.com/tissuestart
		2 injection nozzles		
Biobot Basic	∼$5.000,00	Syringe-based extrusion	United States	https://www.advancedsolutions.com/biobot-basic
		1 injection nozzle		
CELLINK Inkredible	∼$5.420,00–9591,81	Syringe-based extrusion	United States	https://3dprintingindustry.com/news/top-10-bioprinters-55699/
BioBots BioBot1	∼$10.000,00	Syringe-based extrusion, blue light technology	United States	https://www.aniwaa.com/product/3d-printers/poietis-ngb-r/
Advanced Solutions’ BioAssemblyBot	∼$160.000,00	Six-axes syringe based extrusion	United States	https://3dprintingindustry.com/news/top-10-bioprinters-55699/
RegenHU’s 3D Discovery + Biofactory	∼$200.000,00	Syringe-based extrusion	Switzerland	https://3dprintingindustry.com/news/top-10-bioprinters-55699/
		4 injection nozzles bioink and osteoink		
Poietis NGB-R	∼$300.000,00	Combines laser-assisted, micro-valve and extrusion bioprinting	France	https://www.aniwaa.com/product/3d-printers/poietis-ngb-r/

Considering these costs and accessibility issues, we describe herein a low-cost open source microextrusion bioprinter viable for use in research centers with limited resources worldwide. The entirety of our design, code, bill of materials and instructions can be found at the following repository: https://github.com/Laboratory-of-Cellular-Communication/E-Waste-3D-bioprinter and can observed the materials used in Table 5. In addition, assembly schematic guideline can be achieved from Supplementary Material.

**TABLE 5:** Prices of materials to build the 3D bioprinter^a^.

Items	Price	Reference
Driver CD or DVD rom	$22,99	https://amazon.com
Threaded screws and Customs	$12,99	http://amazon.com
Acrylic sheet 5 mm thickness	$18,99	http://amazon.com
Urinary catheter	$18,99	http://amazon.com
BD disposable luer lock syringe	$0,20/syringe	http://fischersci.com
BD general use and precisionglide hypodermic needles	$0,25/needle	http://fischersci.com
Arduino mega 2560 board	$48,00	http://amazon.com
Allegro A4988 module	$2,04/item	http://amazon.com
Hiletgo 2 items a4988 v3 compatible cnc shield^b^	$7,99	http://amazon.com
Omron ee-sx1235a-p2	$2,95	http://octopart.com
Infusion pump	^c^	
Total price	∼$135,65	

^a^ These prices also include material acquisition to build 3Dbioprinter. The final cost will depend on how much material is obtained as E-waste.

^b^ This price includes an *arduino* board coupled CNC, shield, which can be lower.

^c^ The Infusion pump used herein was found a hospital waste. Some open-source infusion pump are available (Wijnen et al., 2014; Booeshaghi et al., 2019).

### 1.1 Extrusion-based methods and their advantages

Microextrusion is the cheapest and easiest to use bioprinting technology, as it can be developed by adding a syringe extrusion system as a print head to commercially available 3D printers. The bioinks are ejected through a microneedle (Panwar and Tan, 2016) alternating the deposition of hydrogel that will serve as the scaffold with deposition of bioink deposited by at second nozzle. This technique can achieve high polymer density, but only moderate resolution (widths ranging from five microns to a few millimeters) and an after-print cell viability ranging from 40% to 80%, which will also depend on the employed bioink type and viscosity (Zhao et al., 2015).

Access to these types of printers is not easy everywhere, as their cost is still high for certain regions/institutions and logistics problems (importing costs, customs paperwork, *etc.*), make the technology difficult to reach outside the global north. Thus, a truly accessible, customizable, and cost-effective bioprinter should be amenable for construction with locally available materials and technologies. The development of a bioprinter employing microextrusion-based bioprint is a necessity and also an opportunity to democratize the teaching of basic technologies.

## 2 Methods

### 2.1 Building the bioprinter

**FIGURE 1:**
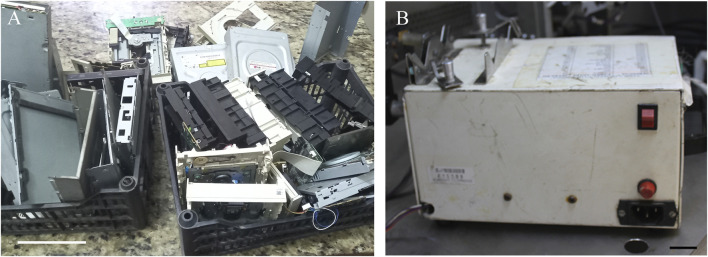
Representative image of the recycled scrap material used to build the scrap metal-based low-cost bioprinter. **(A)** CD, DVD drivers, and acrylic pieces. Scale bar: 10 cm. **(B)** Injection subsystem: Representative images of the equipment used for the injection subsystems within the extrusion system. Scale bar: 2 cm.

To develop the printer, a Cartesian movement system (three orthogonal axes) was employed. Sourced material was obtained from scrap metal (Figure 1A). Based on available online repositories (DIY Micro Dispensing and Bio Printing, 2022), CD, DVD and acrylic pieces, as well as threaded metal columns, several screws and small metal “L” angle brackets, were used to create a 3D positioning subsystem mechanical part.

**FIGURE 2:**
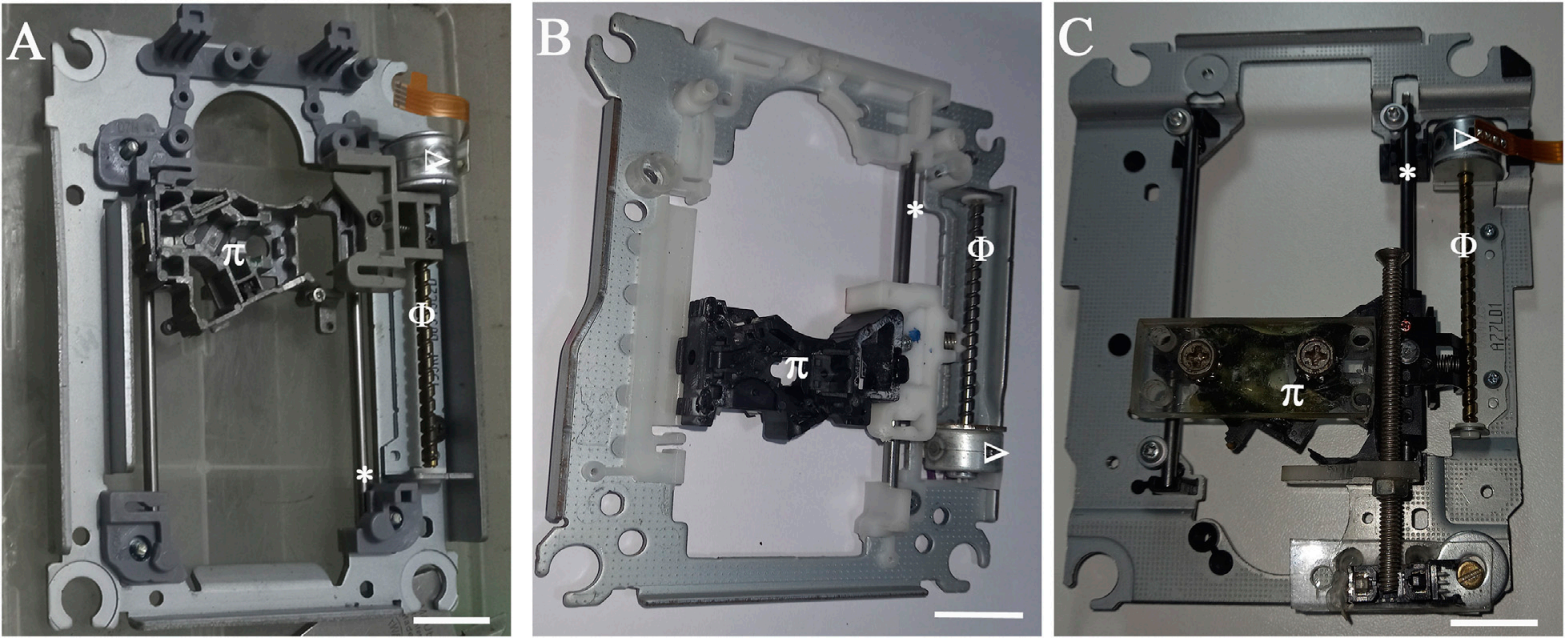
Mechanical blocks to construct the three orthogonal axes. **(A)** X-axis driver set **(B)** Y-axis driver set **(C)** Z-axis driver set. Φ: Worm Thread; *: linear guides; Δ: stepper motor; Π: optical assembly support. Scale bar: 2 cm.

**FIGURE 3:**
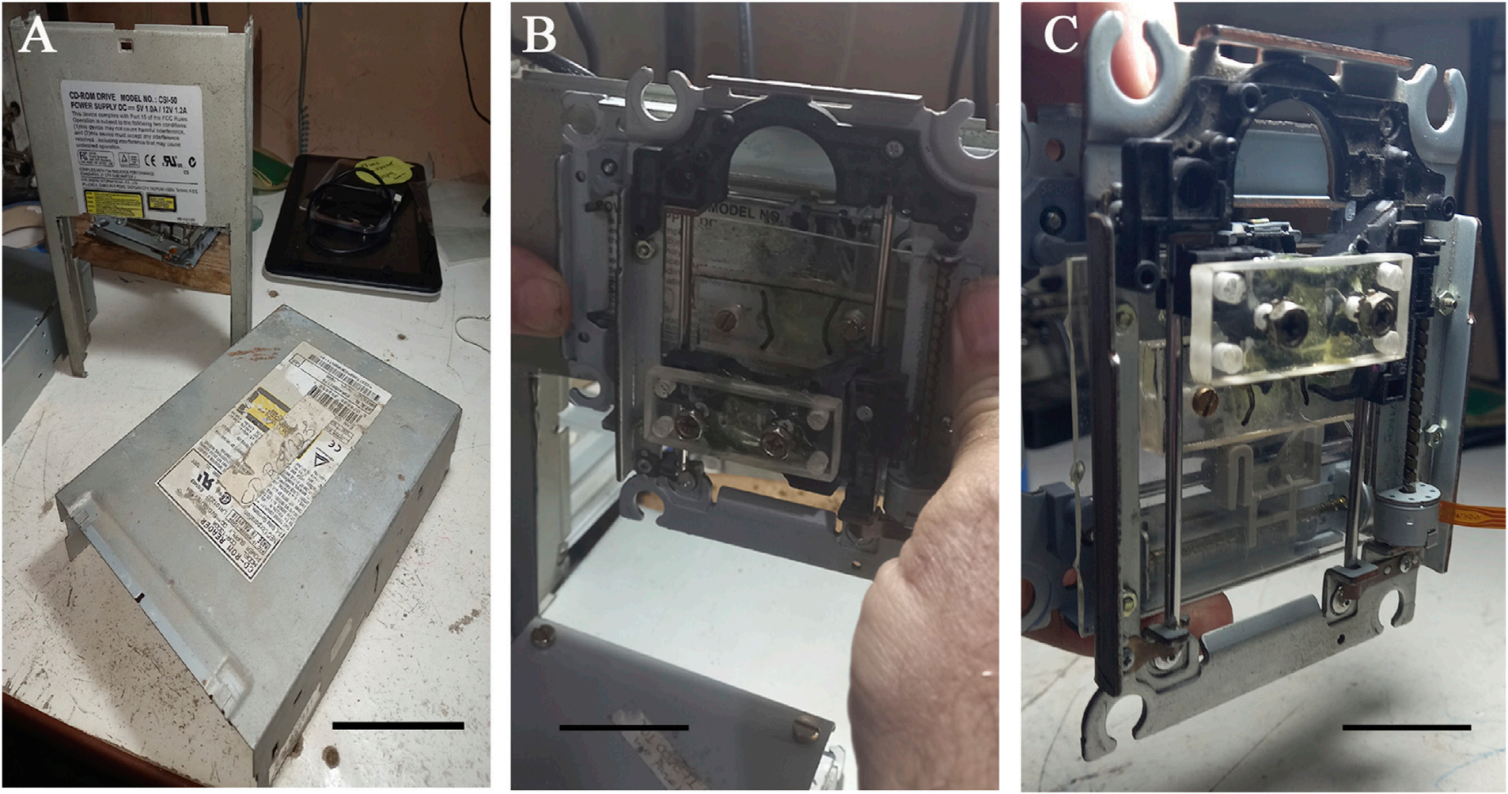
Mechanical setup of the axes in the 3D movement block. **(A)** Steel cover plates from CD-ROM boxes used to prepare the main 3D printer structure. A window was cut in the vertical plate to allow for the Y-axis movement. Scale bar: 5 cm. **(B)** Setup of the X-axis block in the upper parts of the vertical plate. An acrylic plate rectangle was glued onto the optical block support of the X-axis to attach the Z block. Scale bar: 5 cm. **(C)** Setup of the Z-axis block with the rectangular acrylic plate glued onto the optical block support of the Z-axis with screws used to attach the needle support. Scale bar: 3 cm.

**FIGURE 4:**
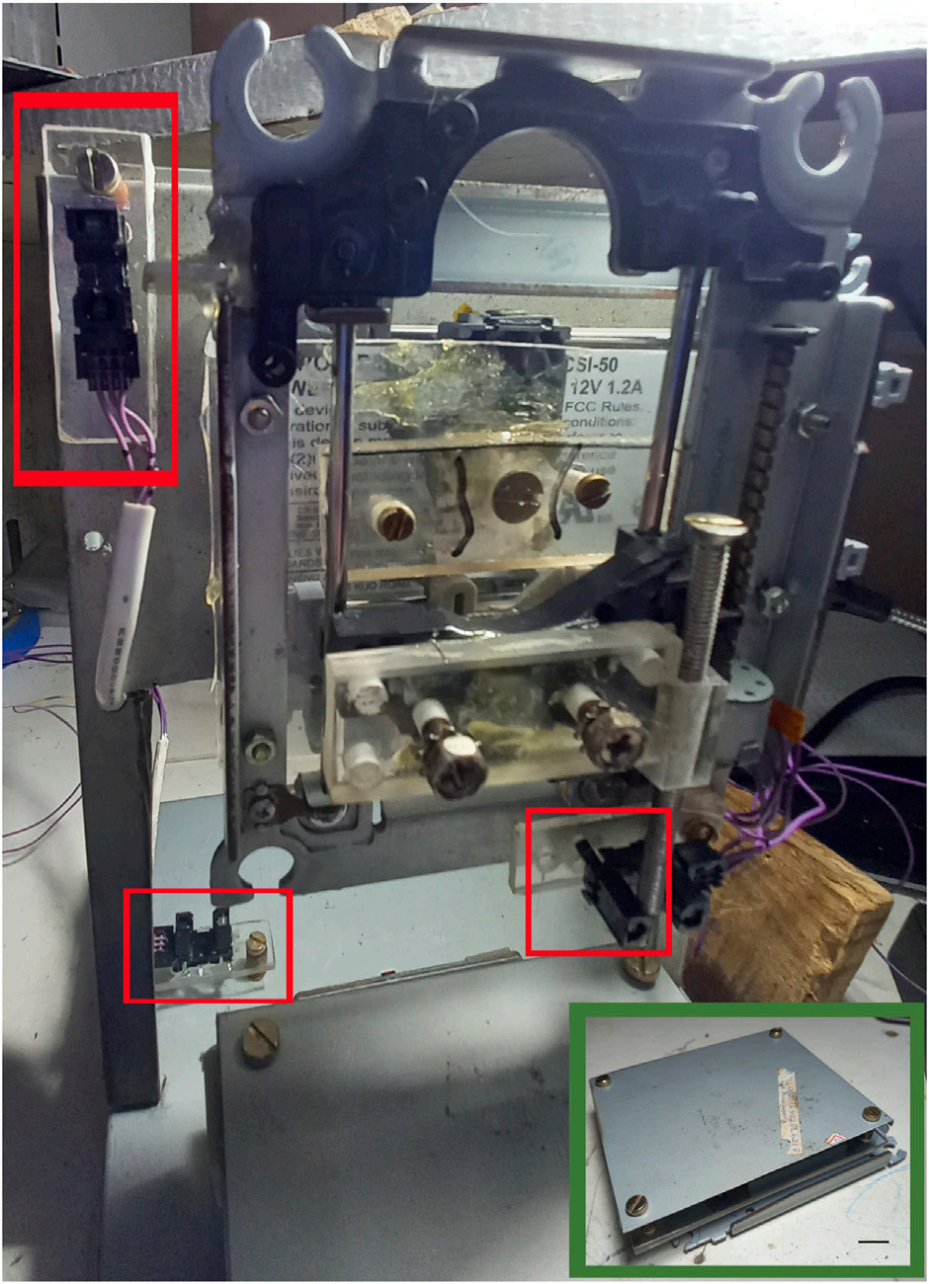
Limit switch installation with locations indicated in red squares. Insert: Mechanical assembly of the printing table. The four screws are used for leveling. Scale bar: 1 cm.

After assembly of the motorized mechanical structure (Figure 2), mechanical axes setup was performed (Figure 3), also including the printing table (Inset Figure 4), the optical limit switches (Figure 3), electronic boards (Arduino and Shield), power supply, wiring and limit switches were installed (Figure 4).

#### 2.1.1 Plunger extrusion subsystem

An infusion pump employing a syringe was used for the injection system. Specifically, a plunger housing, transformer, spindle and locking end-of-stroke sensors (Figure 1B) were used. It is important to note that using this pump in the injection system brings in an extra cost to the bioprinter, due to the difficulty of accessing this as scrap/recyclable material (e.g., hospital disposal). Even so, our project remains more accessible than other available solutions. Otherwise, there are some open-source pump project available that may be used in this case (Wijnen et al., 2014; Booeshaghi et al., 2019).

#### 2.1.2 Building procedure

**FIGURE 5:**
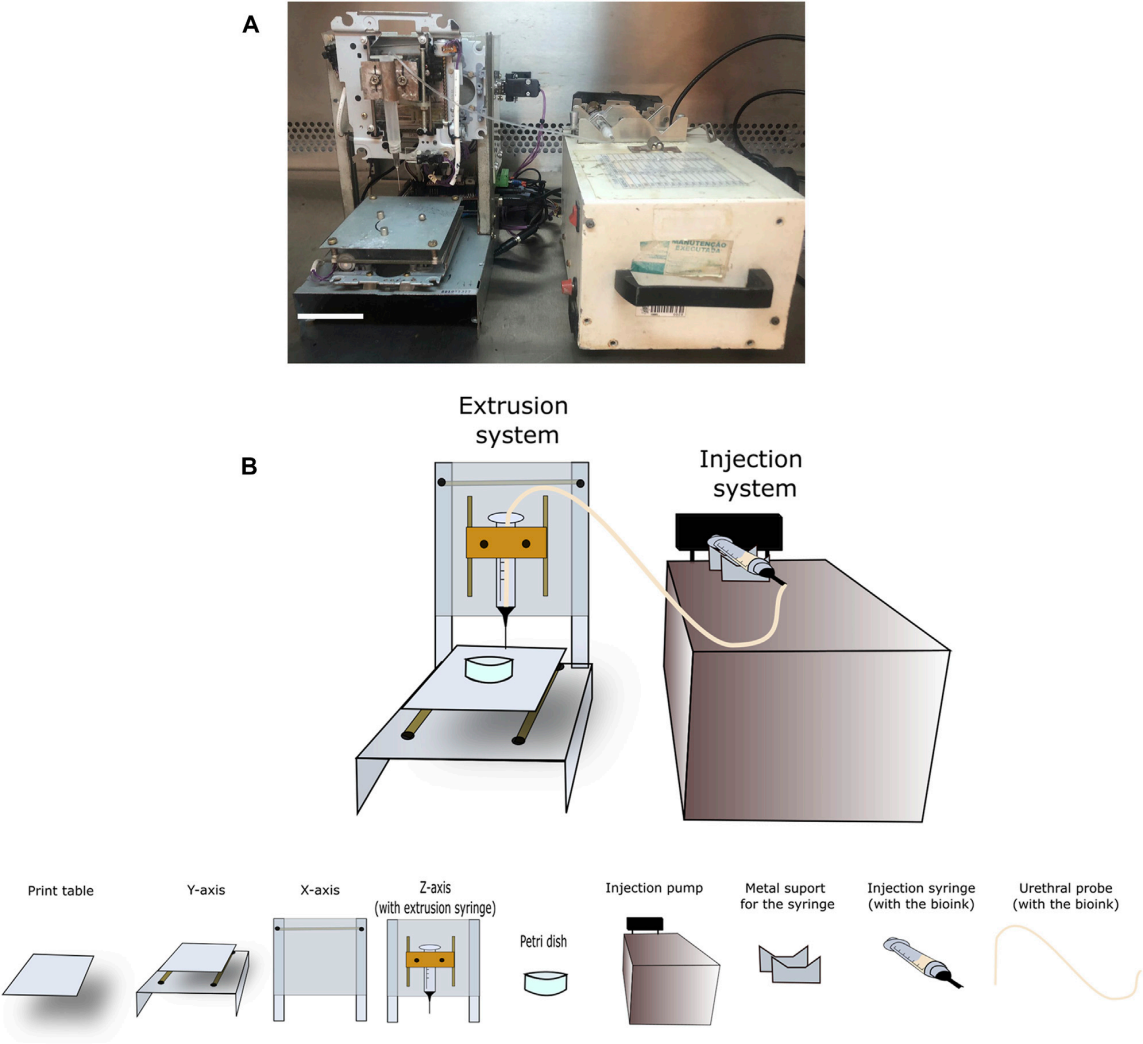
Prototype design. **(A)** Frontal bioprint view indicating all prototype parts, following a **(B)** schematic design indicating details at the bottom describing the infusion pump, table, and extrusion head, as well as the X, Y, and Z-axes. Scale bar: 3 cm.

The representative image of all the components of this bioprinter is depicted in Figure 5 as a schematic design. In order to begin building the bioprinter injection subsystem, an insulin syringe needle was cut to 3 mm from the end of the barrel using a micro grinder diamond-cutting disc, while the 1.2 mm diameter hypodermic needle was cut at a 30 mm distance from the barrel. Both cuts were made to remove the bevel. The plastic hose of the urethral tube was cut to a length of about 32 cm, removing the fitting and the end. The end of the protective cap was coaxially drilled using a 2 mm diameter drill. Subsequently, one end of the plastic hose was inserted into the 1.2 mm diameter needle until the barrel and tied using a few turns of steel wire, tight enough so that the hose would not slip on the needle. The lashing, the end of the cannon and part of the tube up to the end of the needle were covered with a thick layer of epoxy glue, not enough to allow dripping. The cap was then inserted, with the hose inserted through the hole until covering the needle. The plunger of the insulin syringe was removed, the rubber separated from the rod and the rubber end of the plunger pierced with the 2 mm drill bit, so opposite end of the plastic hose is inserted into the hole from the side of the rod fitting. The end of the hose lines was joined with the rubber end of the plunger.

The plunger rubber was inserted into the syringe to about 1 cm below the handle flange. The space to the flange was filled with epoxy glue (∼0.2 mL) and small balls of soft paper (∼3–4 mm in diameter) were inserted between the plastic hose and the syringe wall, filling the space around the hose to act as a plunger for the glue. The assembly was pushed until the rubber plunger touched the bottom of the syringe, to minimize the volume between the hose opening and the syringe needle.

#### 2.1.3 Electronic modules

**FIGURE 6:**
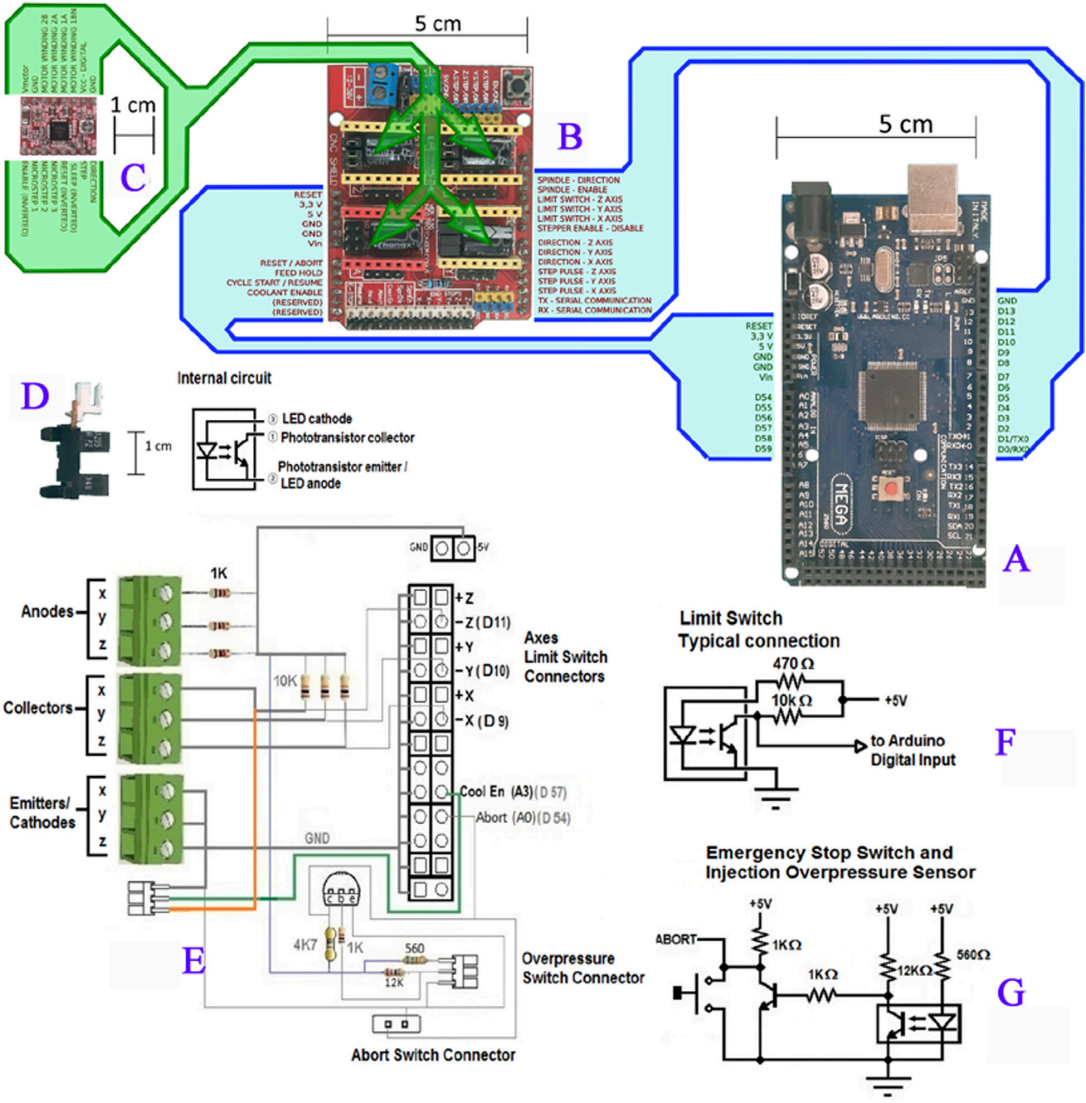
Prototype electronic components. **(A)** Arduino Mega 2560 board, with the respective pin out identification (id. absent for unused pins). **(B)** CNC Shield board for Arduino Uno/Mega, with the function name of the pins in the same respective order of the corresponding pins in the Arduino. **(C)** Stepping motor Allegro A4988driver module, with the corresponding functional pin identifications. **(D)** Transmissive photomicrosensor OMRON EE-SX1235A-P2 used as the limit switch. Left: side vision of the photomicroswitch, where the block in the upper right part of the picture, above the gap, is the phototransistor and the block in the lower right is the LED. Right: Schematic diagram of the sensor. **(E)** Wiring layout drawing of the limit switch connections. **(F)** Typical photosensor connection as the limit switch, used for the X, Y, and Z-axes and for the Injector (named “A axis” in the CNC board labels). **(G)** Circuit schematic of the overpressure sensor and Emergency Stop Switch.

The electronics were based on the Arduino Mega 2560 board (Figure 6A), with the respective pinout identification (id. absent for unused pins). The stepping motor driver module used was the Allegro A4988. The CNC shield board contains an area to install four of these modules, one for each axis and one for the bioink injector (Figure 6B). The motor connector are the four horizontal pins bellow each module (Figure 6C). The transmissive photomicrosensor OMRON EE-SX1235A-P2 has a limit switch and a photomicroswitch and phototransistor (Figure 6D). The block in the lower right is the LED. The overpressure sensor and emergency stop switch are connected in parallel to the “ABORT” digital input of the CNC shield board. When one of these switches goes to the “LOW” logic level, the software immediately interrupts the printing process Figure 6E–G. The overpressure sensor is a microswitch connected to the injector mechanism. In case of obstruction of the bioink flow, the increased pressure inside the syringe exerts an axial force in the mechanism and produces a small displacement of the driver screw against a spring, enough to press the microswitch and abort the printing, avoiding damages in the injection system due to excessive forces.

#### 2.1.4 Software and the performer parameterization

After constructing and testing the bioprinter mechanics, we created digital models of test parts using Wings3d (http://www.wings3d.com) or Tinkercad free web app (https://www.tinkercad.com/). These were exported as STL (Standard Tessellation Language) files and imported into RepetierHost (https://www.repetier.com/), which both controls printer movements and contains the Slic3r (https://slic3r.org/) module, used to transform STLs into the Gcode, the series of commands that will be interpreted by repetierHost to move the printer sequentially, following the model initially created and extruding bioink. The Y and X-axis parameterized with a one-step 10 µm resolution with a maximum non-printing move speed of 80, usual printing speed range of 2–10 and 36 mm axis range. In addition, the *Z*-axis has a step resolution of 10 μm, 40 mm/s of maximum non-printing move speed, a usual speed rage of 2–10 and 20 mm axis range.

### 2.2 Preparing to print

#### 2.2.1 Decontamination

a. To minimize contamination, the bioprinter is placed inside a hood, and subjected to at least 30 min of UV light decontamination prior to use.
b. Peracetic acid solution is used to decontaminate the pathway in which the bioink is strained for bioprinting. The probe is soaked in a 0.2% peracetic acid aqueous solution for 10 min prior to use. The probe is then washed three times with sterile deionized water.

#### 2.2.2 Making and printing designs

We prepared four models for this project, a 5 mm × 5 mm x 2.5 mm (L × W × H) cuboid block, 3D, heart, and star shaped model. The first model was designed in Wings3D and the others were drawn in Tinkercad, exported to STL and imported into repetierHost, where they were scaled and sliced into G-CODE using the Slic3r module.

#### 2.2.3 Developing a bioink formulation

In order to verify the accuracy of our prototype, we printed a construct with pluoronic-F-127 (30%w/v) (#SLBG6026V, Sigma, United States) without cells and with the addition of an orange food coloring to improve visualization.

##### 2.2.3.1 Alginate with CaCl_2_ 30 mM

i. A 6% sodium alginate solution (4% w/v) (W201502-1 KG, #MKBX3379V, Sigma, United States) was prepared in 100 mL of 0.9% w/v saline solution (Sorimax, FARMAX). Stirring was maintained throughout the preparation. The temperature was maintained at around 60°C to completely homogenize the alginate.
ii. A 3-fold higher calcium chloride (CaCl_2_) (C50080-500G, #083K0037, Sigma, United States) concentration was prepared to maintain the final solution concentration. A total of 0.03 g of CaCl_2_ were mixed with a 0.9% w/v saline solution, resulting in a 60 mM solution.
iii. Solutions I and ii were mixed at a 3:1 ratio, resulting in a 4% sodium alginate solution containing 30 mM CaCl_2_. The solutions were then autoclaved and put to use.
iv. 1 mL of the bioink was drawn with a 3 mL sterile syringe (990174, BD Luer-LokTM Tip, BD PlastipakTM) and placed in the bioprinter to begin the printing process.
v. To complete polymerization after printing, 500 µL of a 5 mM CaCl_2_ aqueous solution was dropped on top of the construct for 3 min and the block was cultured at 37°C under a 5% CO_2_ atmosphere in an oven.

##### 2.2.3.2 Alginate and gelatin

i. A 4% sodium alginate solution (Sigma, United States) (4% w/v) was prepared in 100 mL of a 0.9% w/v saline solution. Stirring was continued throughout the preparation. The temperature was maintained at around 60°C for complete alginate homogenization.
ii. A total of 3 g or 4 g of gelatin (G8150-100G, #113K0029, Sigma, United States) were mixed with the solution mentioned above to prepare a 4% sodium alginate solution containing 3% or 4% gelatin solution, respectively.
iii. Since this bioink seems ideal to bioprint, we added cells to the printed construct assess biocompatibility. Around 10^7^ cell/mL HEPG2 lineage was bioprinted, as mentioned previously, on a 35 mm × 10 mm Petri dish.
iv. To complete polymerization after printing, 500 µL of a 5 mM CaCl_2_ aqueous solution was dropped on top of the construct for 5 min and the block was cultured at 37°C under a 5% CO_2_ atmosphere in an oven.

###### 2.2.3.2.1 Caution ▵!.

It is important to note that all solutions can be stored for 1 month after preparation at 4°C. After this, printability may be compromised.

##### 2.2.3.3 Preparing the bioink with bioprinting cells

i. Bioink temperatures of both the alginate and calcium chloride and alginate and gelatin solutions must be used at around 37°C, as cells are maintained at that temperature.
ii. The bioinks were placed in a water bath to complete melting, at around 57°C. After that, we wait for temperature decreases at 37°C. Cells at the concentration of interest were washed and placed in a polypropylene tube. A total of 1 mL of the bioink was added slowly to avoid bubbles and then placed in a 1 mL syringe.
iii. After placing the bioinks in a syringe, they were maintained at 4 °C for 1 h to improve polymerization.
iv. A total of 500 µL of calcium chloride were added to the top of the blocks for 3 or 5 min, as described previously. The calcium chloride solution was removed with the aid of a pipette and 3 mL of high glucose DMEM (Sigma, United States) medium supplemented with 10% fetal bovine serum (FBS - GIBCO, United States) was added. The culture was performed as described previously.

### 2.3 Progressive cavity pumping extrusion subsystem: Crosslinking agent nebulization subsystem

**FIGURE 7:**
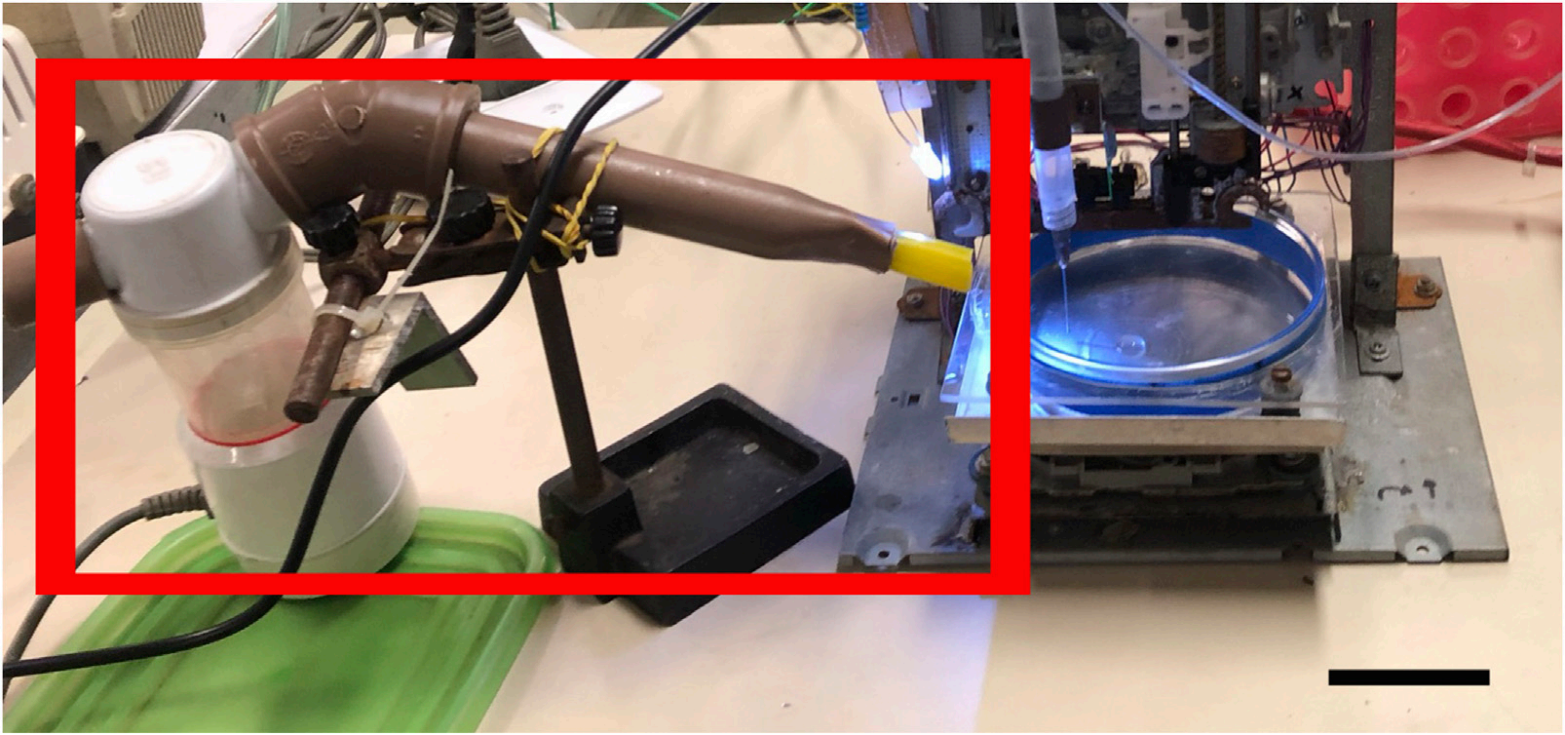
Calcium mist with a nebulization device. Nebulization device (red square) coupled to the bioprinter equipment to solve submerged calcium chloride printing issues. Scale bar: 3 cm.

In order to improve print quality and resolution, an automated calcium chloride nebulization system at different concentrations was combined (Figure 7), providing a jet of this mist on the gelled alginate at the end of the print (Raddatz et al., 2018), since the alginate required ions^2+^ for ideal gelification. However, this system caused clogging at the end of the extrusion needle and led us to abandon its use altogether. Other modifications can also improve alginate gelation, such as association with gelatin or collagen, both biocompatible materials, promoting higher bioprinted material viscosity and better resolution (Andersen et al., 2015).

## 3 Results

### 3.1 The first bioprinting steps: Initial print tests

The materials used to carry out the first calibration injection tests were sodium alginate at 100 mg/mL and calcium chloride at 5 mM. It is important to note that calcium chloride can improve alginate gelification while being biocompatible, maintaining cell viability. In order to better visualize the alginate print in these first tests, a purple food coloring was added.

**FIGURE 8:**
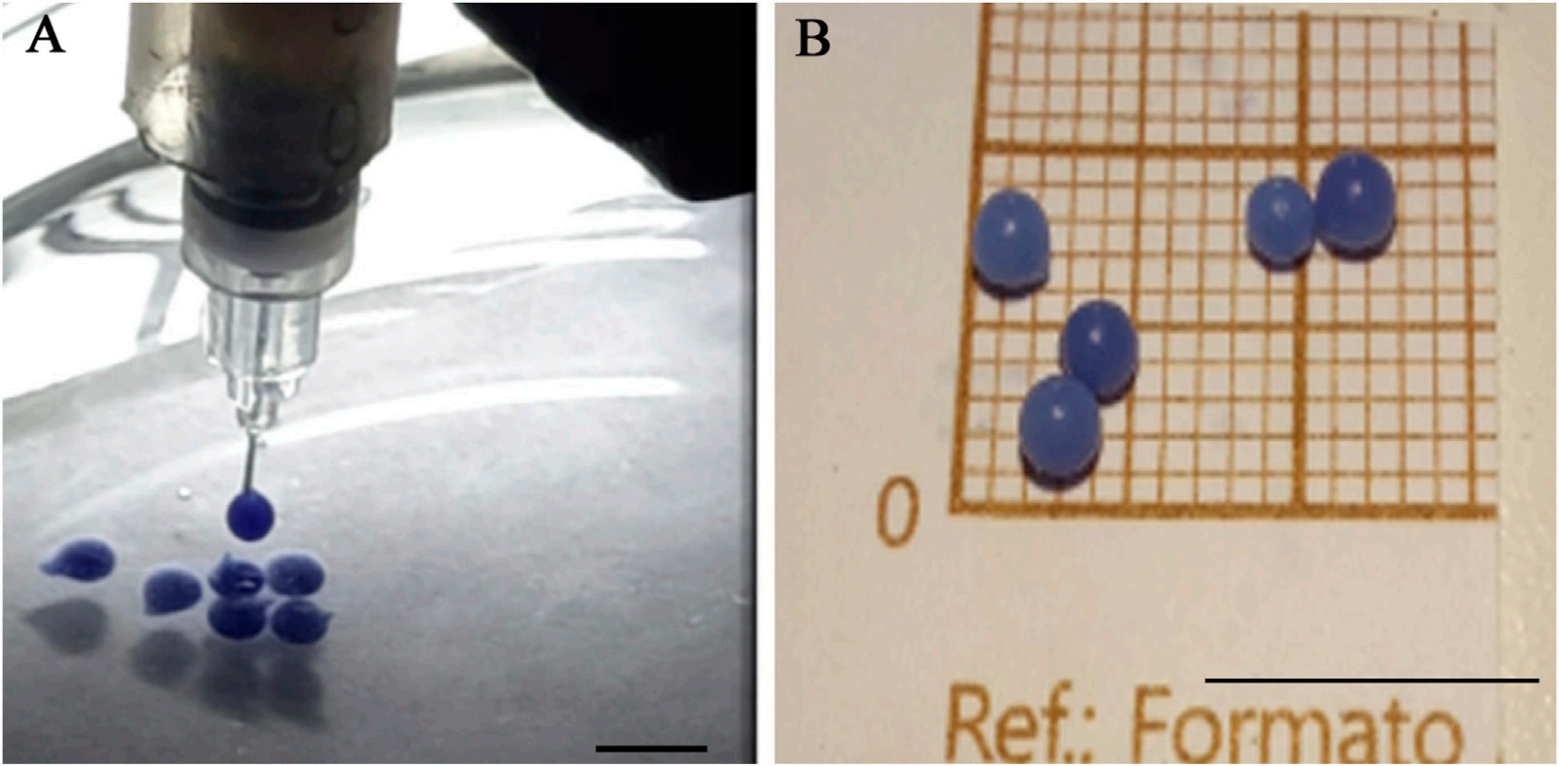
Alginate gelification in calcium chloride: **(A)** dripping and **(B)** appearance of the gelled capsules. Scale bar 1 cm.

The first verified parameter comprised alginate gelling efficiency. Dropping alginate into the calcium chloride solution directly from the extrusion needle produced stable and mechanically resistant-gelled alginate capsules approximately 2 mm in diameter (Figure 8).

#### 3.1.1 Caution ▵!

**FIGURE 9:**
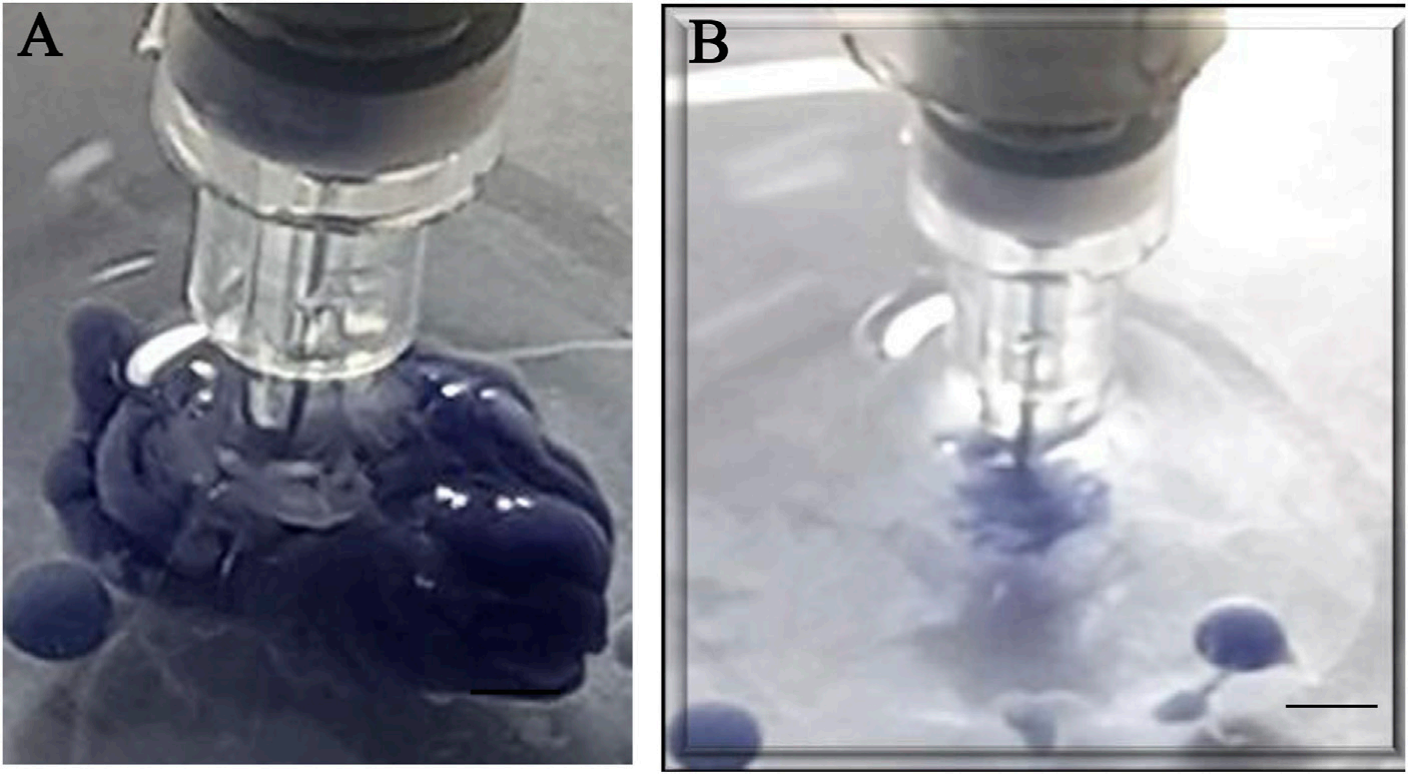
Representative images of the problems caused by alginate gelification insufficiency: **(A)** Excess extruded alginate, **(B)** Lack of extrudated alginate due to needle stickiness. Scale bar: 1 cm.

The first tests indicate some issues, as follows (Figure 9).

- Excess amount of extruded alginate;
- Problems with Petri dish alginate adherence, with the alginate remaining attached to the injection needle;
- Low precision of the table adjustment to needle movement coordinates.

**FIGURE 10:**
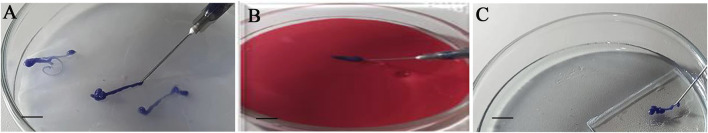
Adhesion tests employing different material surfaces. Representative images of different materials used to verify alginate adhesion to surfaces from extrusion bioink. **(A)** Tracing paper, **(B)** Polypropylene and **(C)** Acrylic. Scale bar: 1 cm.

Several materials were tested to solve glass adherence issues, such as tracing paper, polypropylene, and acrylic (Figure 10), observing the contact angle. The best adherence material was glass with smaller contact angles. The test was then carried out immersed in a calcium chloride solution with the needle tip moving to check for adhesion or trailing.

#### 3.1.2 Caution ▵!

Alginate adhesion to the different surfaces under calcium chloride immersion was not satisfactory, so the printing was not performed immersed. It is important to note that calcium chloride in gelled samples can serve as a source of calcium for ungelled alginate (Andersen et al., 2015). Although adhesion took place, the formed layers were not stable, possibly due to insufficient calcium diffusion, with part of the printed alginate not gelling and flowing, blurring the printing. A calcium mist test was also performed (Figure 7).

#### 3.1.3 Caution ▵!

During this step, the calcium mist minimized the adherence caused by the immersion printing, although the needle still suffered from lumps formed by alginate gelation when extruded.

#### 3.1.4 Caution ▵!

Gelled alginate lumps were observed at the end of the injection needle at the lowest calcium chloride ratio. As these lumps increase in size, they spread the deposited alginate and ink the printing, in addition to obstructing nozzle of injection and, occasionally, accumulating alginate.

The lumps increased as the alginate was attracted by capillary action to the needle. The cannula wall, thus, was coated with a protective hydrophobic membrane (e.g., solid vaseline).

#### 3.1.5 Caution ▵!

The hydrophobic protection, assigned by vaseline also caused calcium chloride repulsion. This can be minimized by adjusting the position of the chloride injection closer to the end of the alginate injection needle.

### 3.2 Print improvement attempts

To improve the printable form, tests with a 3:1 alginate to calcium chloride ratio were performed, and an insufficient amount of calcium chloride was observed. Thus, the chosen model by the Wings3D builder for the tests presented in this report was a standardized 5 × 5 × 2.5 mm block.

Subsequently, 1:3 and 1:1 alginate to calcium chloride ratios were tested, but excess calcium chloride was noted through higher alginate polymerization and the presence of two bioink phases, making material printing impossible. Thus, the first test proved to be the best ratio.

**FIGURE 11:**
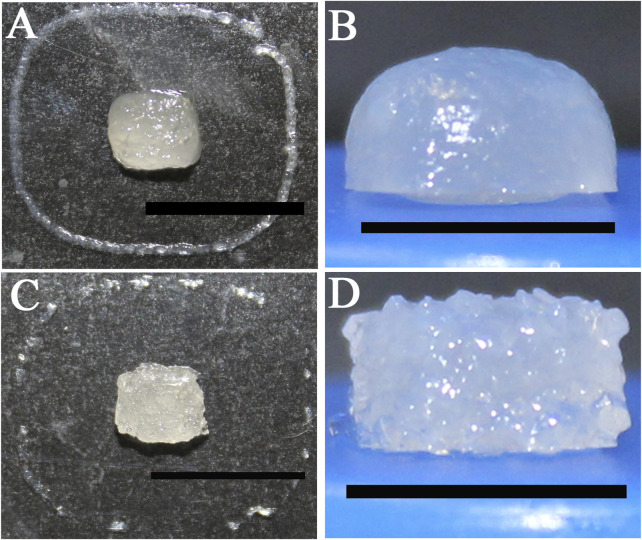
Construct bioprinted with a mix of biopolymers at different concentrations. Bioink comprising 4% alginate and 3% gelatin **(A,B)**, Bioink comprising 4% alginate, 4% gelatin and post-printing crosslinking with 5 M calcium chloride **(C,D)**. Scale bar: 1 cm **(B,D)**; 2 cm **(A,C)**.

**FIGURE 12:**
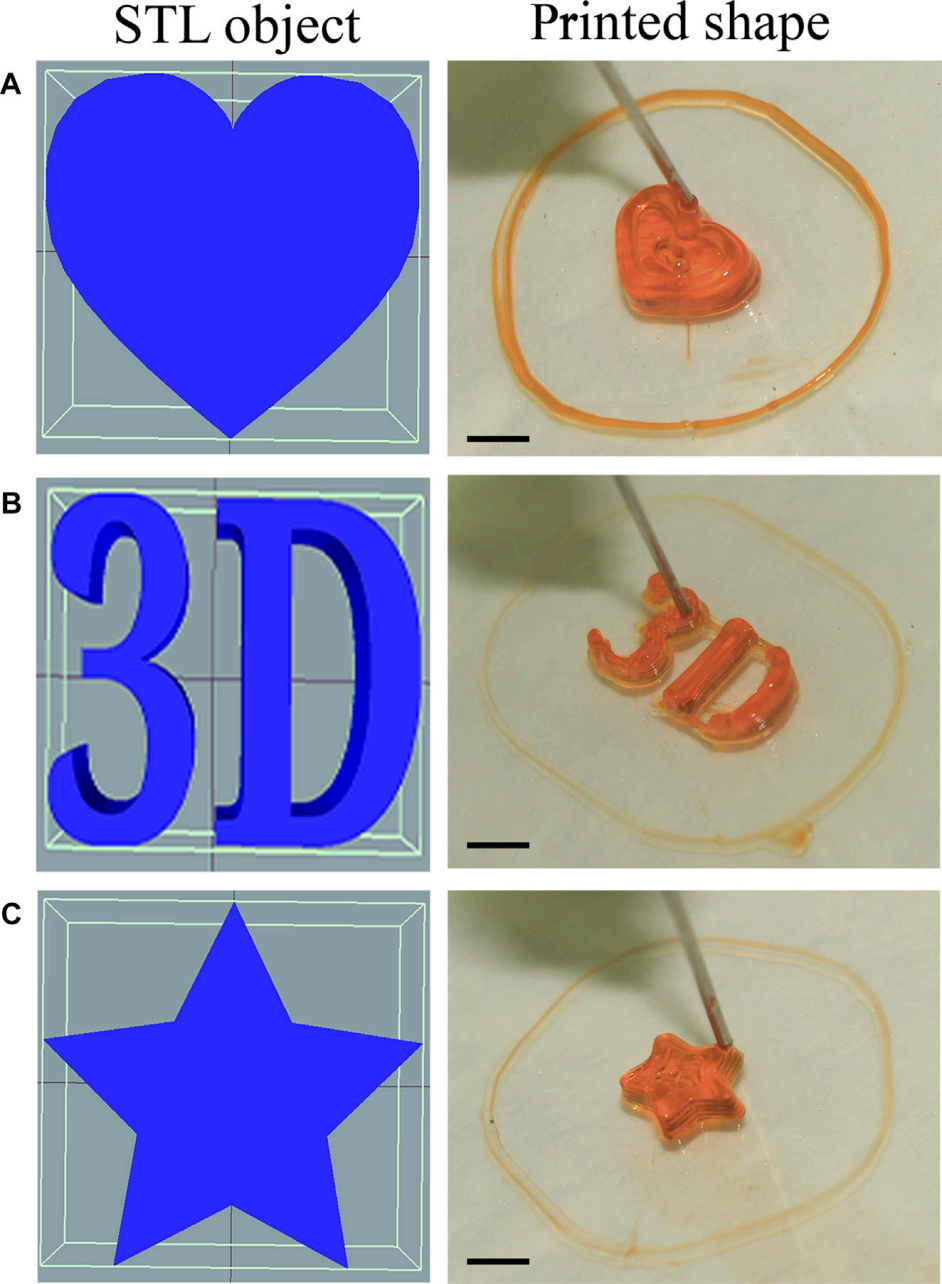
Accuracy test for resolution of the constructs printed by the bioprinter employing the following: Shape printed with Pluoronic-F127 15% w/v without cells and employing food coloring. **(A)** Heart-shaped print dimensions 3 mm × 6 mm × 8 mm **(B)** 3D-shaped print dimension 3 mm × 13 mm × 6.95 mm **(C)** Star-shaped print dimensions 2 mm × 6.18 mm × 7 mm. Right: rendering of models imported into RepetierHost, and Left: Printed shape from our prototype. Scale bar 1 cm.

Printing with alginate and calcium chloride at a 3:1 ratio led to better gelation, but loss of format integrity. Thus, tests employing different concentrations of gelatin and alginate were carried out. The best compositions were 4% alginate with 3% gelatin or 4% alginate with 4% gelatin (Figures 11A, B and Figures 11C, D, respectively), providing the perfect viscosity for bioprinting. Some shape-constructs were also printed using Pluronic-F127 (#SLBG6026V, Sigma, United States) and orange food coloring only, serving as a test for extrusion accuracy for more complex shapes (Figure 12A–C).

### 3.3 Cell viability after bioprinting

**FIGURE 13:**
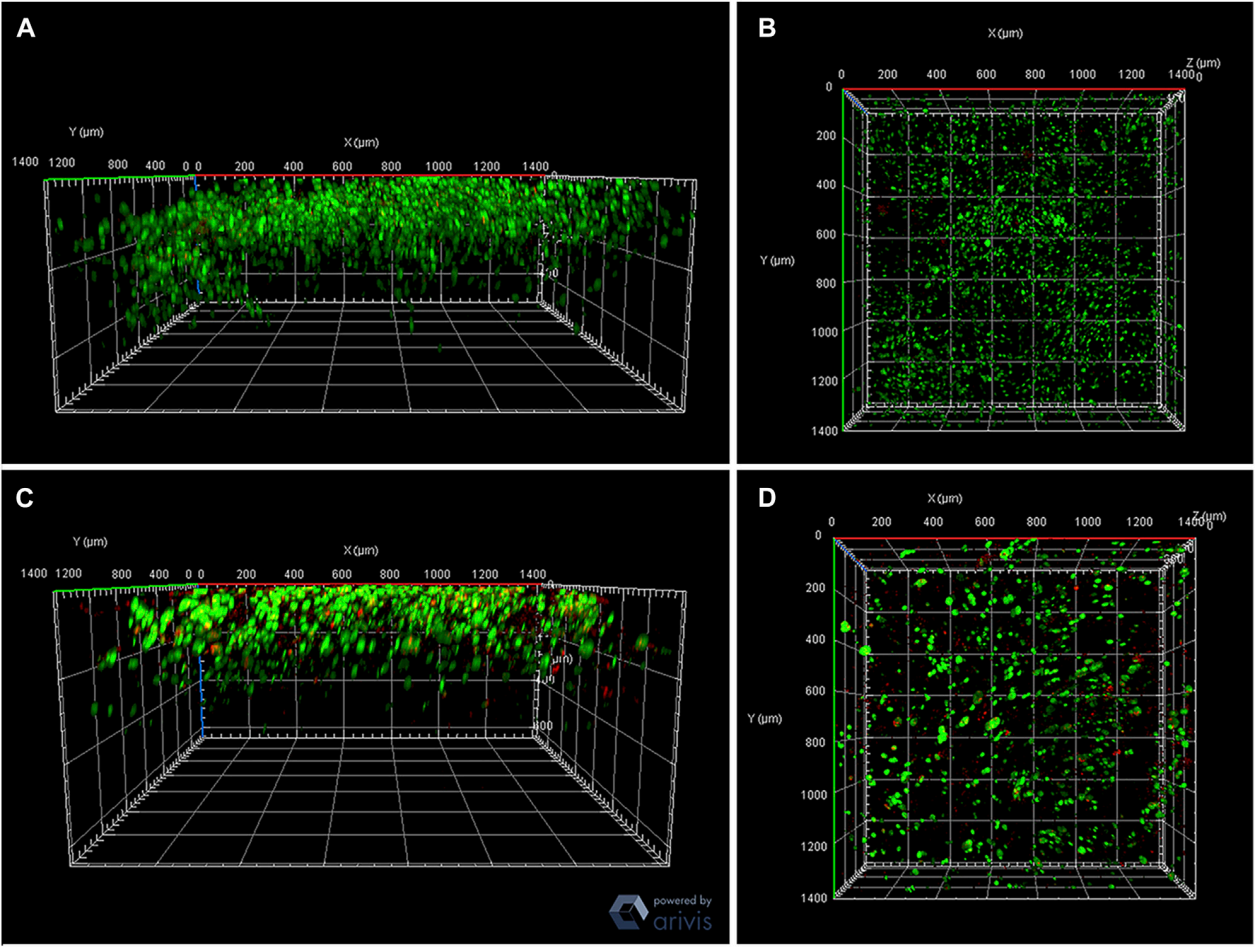
Analysis of extrusion influence on bioprinted construct contains cells. Bioprinted constructs employing alginate 4% and gelatin 3% on day 0 **(A,B)** and in culture after 7 days **(C,D)** after the bioprinting process. Green: Calcein AM; Red: PI (Propidium iodide). View of Z stacks—AxioObserver Zeiss microscope.

**FIGURE 14:**
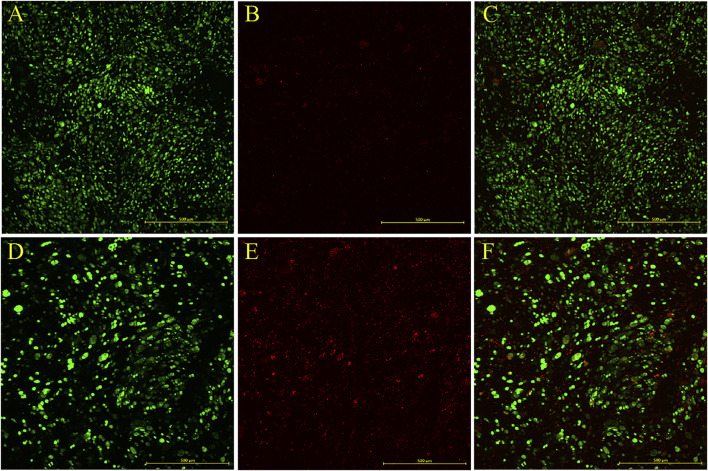
Bioprinted construct viability and maintenance: 4% alginate and 3% gelatin with 107 cells/mL of the HepG2 bioprinted construct on day 0 **(A–C)** and in culture after 7 days **(D–F)**. Green: Calcein AM; Red: PI (Propidium iodide). AxioObserver Zeiss microscope. Construct size: 1.42 mm × 1.42 mm—**(A–C)** Z stack: 129 slices (640 μm) and **(D–E)** Z stack: 124 slices (356.21 1 μm). Scale bar: 500 µm.

After ideal bioprint and a bioink parameterization, cells were added to the printed material. The 4% alginate and 3% gelatin formulation bioink was used in this step. Cell viability analysis was performed employing microscopy to demonstrate that cells were viable right after and 7 days after printing. The fluorophores Calcein/AM and Propidium iodide (PI) were used as viable and non-viable cell markers, respectively, were observed under a confocal AxioObserver Zeiss microscope employing the 3D view ZenBlue software tool and one field Z stack (Figures 13, 14, respectively).

#### 3.3.1 Caution ▵!

Due to the height of the samples, it is difficult to visualize the entire construct fluorescence. The fewer the fluorescent cells, the harder to observe, which is why the PI fluorescence is almost non-existent. Serial cuts of these constructs could be performed to build a broader image.

#### 3.3.2 Caution ▵!

The confocal images indicate that the PI fluorescence is stronger on day 7. Further analyses are required to confirm that cultured cell viability decreases through time.

## 4 Discussion

3D bioprinters comprise cutting edge technology and will certainly become a tool for the improvement of several clinical procedures as therapeutic approaches. However, they are still expensive, keeping them from being broadly adopted and accessed by labs and hospitals worldwide. It is important to note that many proposed low-cost models are high maintenance (Tong et al., 2021). We proposed the construction of a really low cost, accessible and open source bioprinter. To this end, it is important to analyze the functioning and mechanisms behind the assembly and execution of this task, comparing them to the most employed bioprinter models.

Extruded-based bioprint is the most common methodology employed in 3D bioprinting, as it is the most printable material and exhibits better cost-benefits in comparison to other systems (Santoni et al., 2022). Conventional 3D printing software determines extrusion by a linear amount of fused filament. Bioprinters, on the other hand, use liquid bioink, which is a challenge, as it becomes necessary to calculate the extruded volume through the syringe plunger movement. These calculations require some testing to be able to pinpoint the correct match from adjustments with alginate and calcium chloride concentrations, which are also affected by different types of extrusion syringes that can be used with these types of printers. Our prototype aims to improve this feature and improve the printing process. In addition, several software can be accessed and used free or paid, such as https://www.autodesk.com/, https://openscad.org/, https://www.freecadweb.org/, and most companies that have developed a bioprinter have their own encrypted design software (Pakhomova et al., 2020), which strongly affects the cost of bioprinters.

Sodium alginate is a biopolymer widely used in 3D bioprinters. Divalent cations such as Ca^2+^ can induce alginate gelification by crosslinking with polymer chains, forming a 3D structure (Abasalizadeh et al., 2020). However, the increase of Ca^2+^ levels in the alginate hydrogel medium can impair the cell survival, decreasing cell viability (Cao et al., 2012). The use of a single bioink component has been proven limited, as it is harder to achieve biochemical and biophysical properties similar to the natural extracellular matrix. Therefore, other biopolymers must be used to guarantee better printability, viability, and construct shape (Kim et al., 2022). To improve these parameters, gelatin was added to the biopolymer mixture. Gao and coworkers suggested that alginate and gelatin ratio is important to maintain biofunctionality and printability properties (Gao et al., 2018). Formulating a bioink ideal for bioprinting requires optimal parameters regarding mechanical properties, printability, and biocompatibility (Gu et al., 2020) in order to promote an efficient, stable 3D printing while also maintaining high cell viability, for this technology to be applied in transplants or even in toxicological analyses.

## 5 Conclusion

The prototype reported herein paves the way for the assembly of low-cost equipment and wide access to laboratories with little funding. Through the acquired knowledge, subsidies were accumulated for the detailing and improvement of the 3D bioprinter prototype project. In addition, the software and equipment designs are both freely available under open source licenses, democratizing access to these technologies. Our proof of concept indicates cell viability at day 0 and day 7. More studies are required to verify how long the constructs remain viable in culture. This project may serve, therefore, as a low-cost bioprinter option and an important tool for *in vitro* bioprinted 3D tissue applied for future transplantations or drug toxicity tests, presenting itself as an alternative to the use of animals in research.

## Data Availability

All data are available in our github repository (https://github.com/Laboratory-of-Cellular-Communication/E-Waste-3D-bioprinter) as well as in this supplementary material of this manuscript.
