# Supplementary material for: Development and implementation of a significantly low-cost 3D bioprinter using recycled scrap material
Source: Front Bioeng Biotechnol. 2023 Apr 7;11:1108396. doi: 10.3389/fbioe.2023.1108396 (PMC10119389; doi:10.3389/fbioe.2023.1108396)
Supplement: Supplementary file 1 [file DataSheet1.DOCX]

Supplementary Material

Development and implementation of a significantly low-cost 3D bioprinter using recycled scrap material

Jaciara Fernanda Gomes Gama ^1†^, Evellyn Araujo Dias ^1†^, Rosângela Marques Gonçalves Aguiar Coelho ^1^, André Maia Chagas ^2,3,4^, José Aguiar Coelho Neto ^1,5^, Luiz Anastacio Alves ^1*^

**^†^These authors contributed equally to this manuscript.**

*** Correspondence:**Correspondence information: Luiz Anastácio Alves, M.D., Ph.D. - Laboratory of Cellular Communication; Oswaldo Cruz Institute, Oswaldo Cruz Foundation. Brazil Avenue, 4365 Manguinhos, Rio de Janeiro (RJ), Brazil. 21045-900. FAX: +55 (21) 25621816 Tel: +55 (21) 2562 1841. E-mail: [alveslaa40@gmail.com](mailto:alveslaa40@gmail.com)

**Assembly guideline to our 3Dbioprinter**

The drawings presented in this guide were made using the software FreeCAD version 0.20, which was complemented with the add-on named "parts library". This add-on contains many ready-made parts that may be used in many projects. In our project, some of these parts, such as screws and Arduino Mega board were used.

In addition, the following parts available in the “Grabcad” library repository (grabcad.com) were used as it is or with some modifications and, all credits to the respective authors are given in the following box:

| **Model name** | **Author** | **link** |
| --- | --- | --- |
| Cannula G21 for Syringe | Scarus,2021 | https://grabcad.com/library/cannula-g21-1 |
| Syringe 3 ml | Kaloyan Georgiev, 2022 | https://grabcad.com/library/syringe-3-ml-2 |
| Arduino Uno CNC Shield | Anirudh Pednekar, 2021 | https://grabcad.com/library/arduino-uno-cnc-shield-1 (only the CNC Shield3.0 model were used) |
| Syringe pump model 2 | Eng. karim samhy, 2021 | https://grabcad.com/library/syringe-pump-model-2-1 |
| Optical-endstop-for-3d-printers | Sergiy Ozymok, 2017 | https://grabcad.com/library/optical-endstop-for-3d-printers-1 |


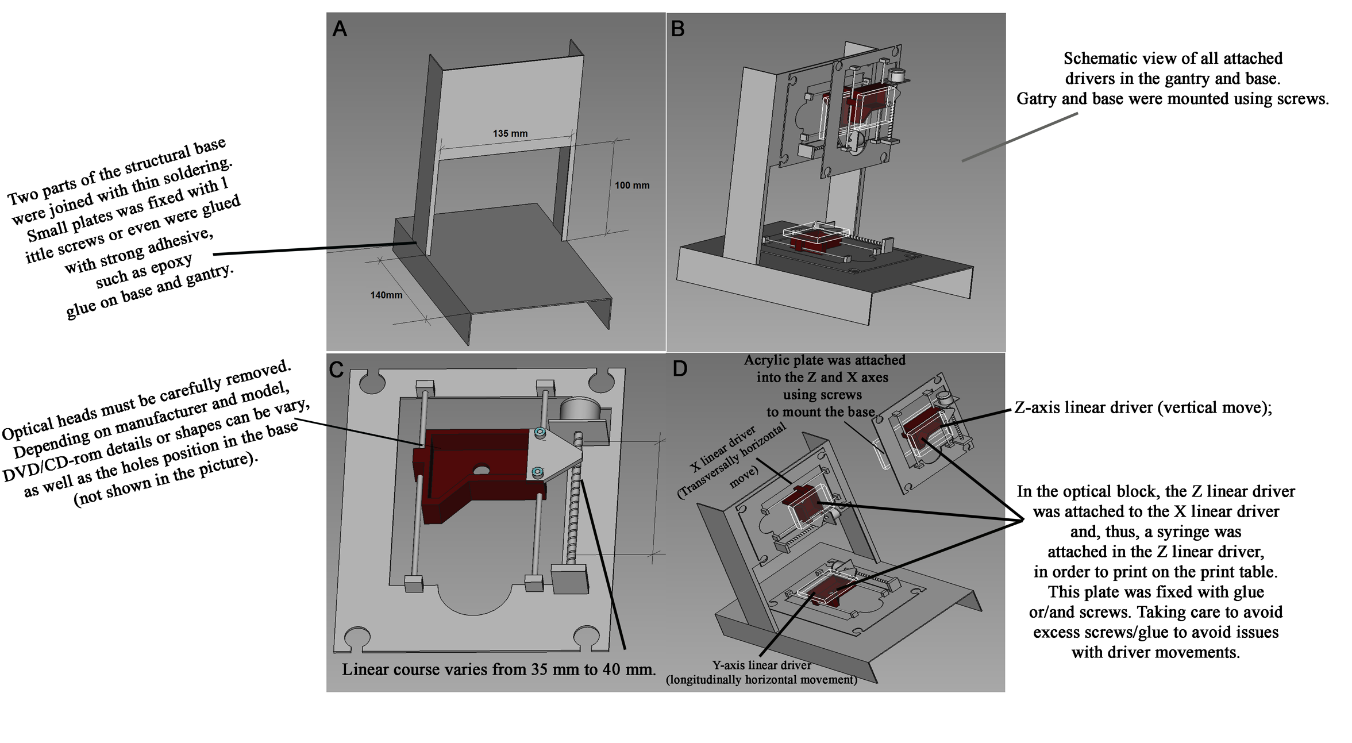


Figure S.1 – First steps to the bioprinter assembly. (A) Base and the gantry mounting; (B) Three drivers schematically overview; (C) Linear course details from used DVD drivers; D – Linear drivers details and attachment.


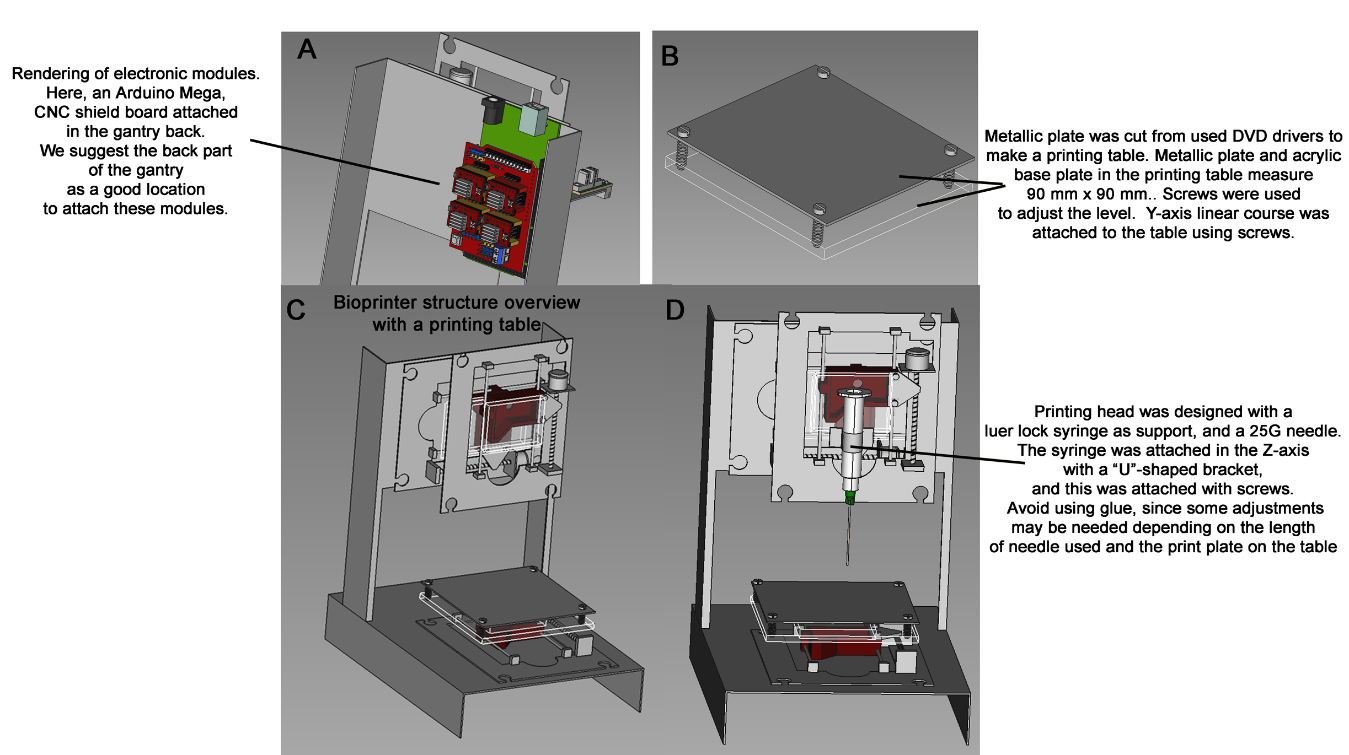


Figure S.2 – Assembly procedure step-by-step. (A) Electronic modules details; (B) Printing table and its base plate details; (C) Printing table attached to de Y cursor overview; (D) Printing head attachment details.

 
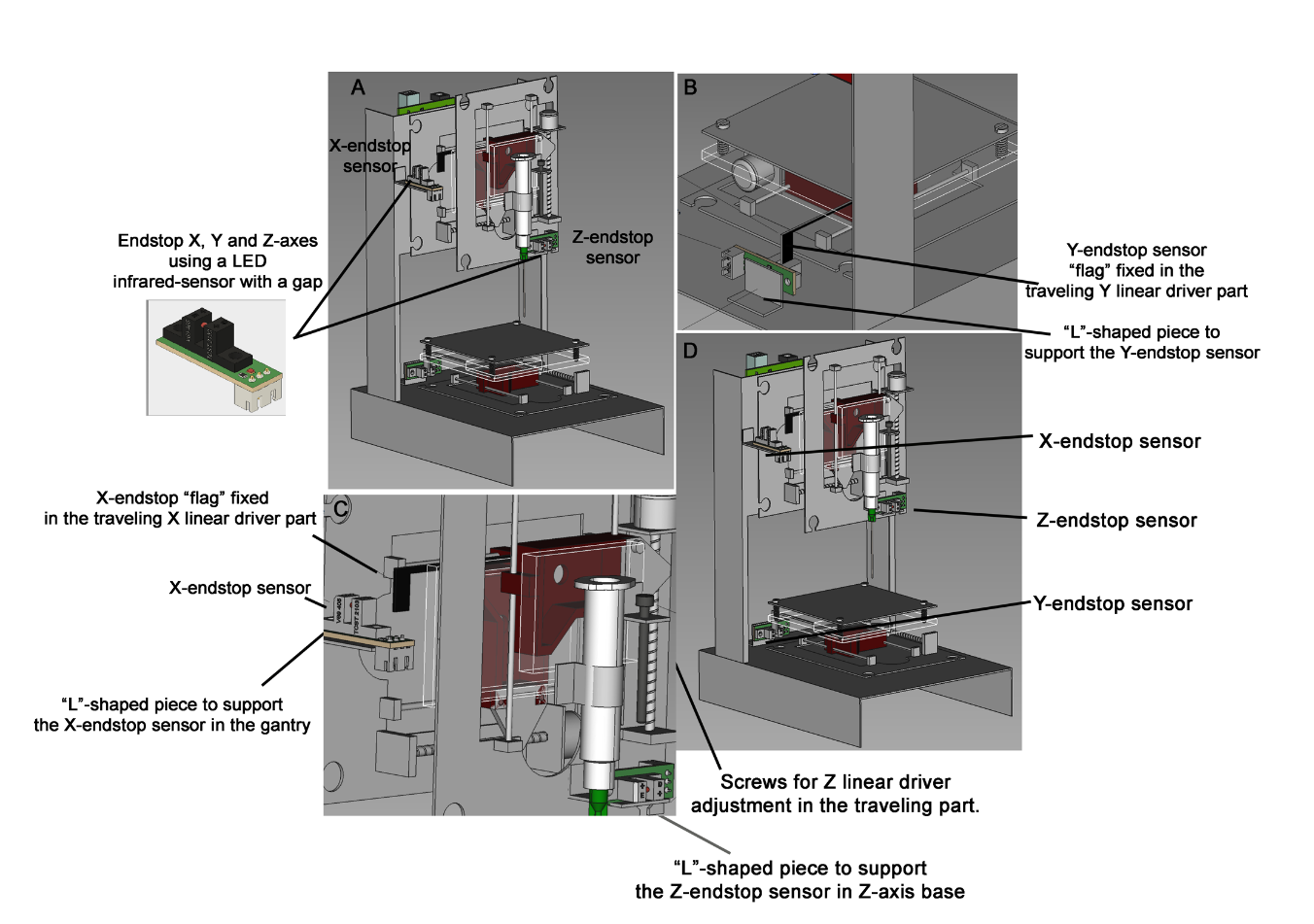


Figure S.3 – Endstop sensors placement. (A) Endstop sensors adjustments step-by-step; (B) Endstop sensor Y assembly; (C) Endstop sensors X and Z assembly; (D) Endstop sensors set overview.


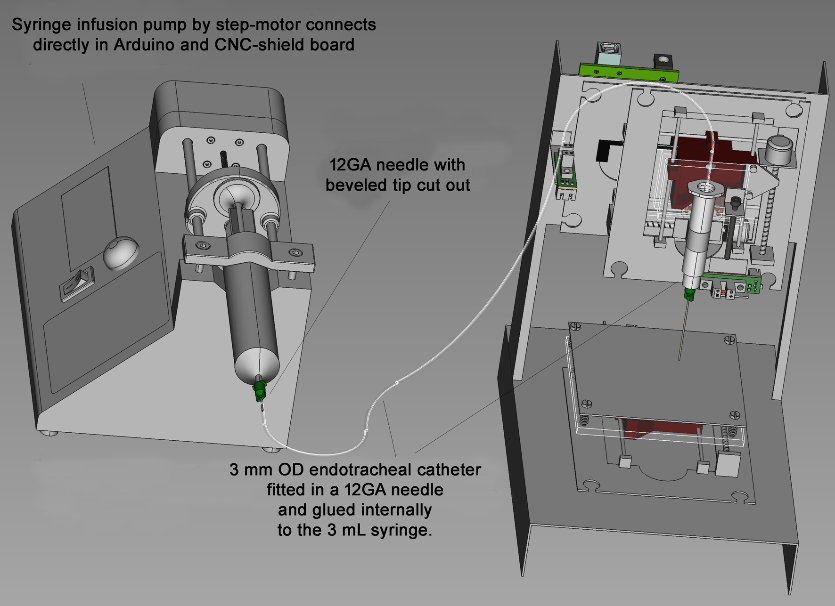


Figure S.4 – Bioprinter schematic overview including injection system details. The infusion pump used in our project was obtained from scrap hospital waste; it is not exactly equal to the pump represented in this figure, however there are some available pump open-source models.
